# Legal Profession and Corruption in Health Care: Some Reflective Realities in South Africa

**DOI:** 10.3389/fpubh.2021.688049

**Published:** 2021-08-25

**Authors:** Evangelos Mantzaris, Pregala Pillay

**Affiliations:** ^1^Mangosuthu University of Technology, Durban, South Africa; ^2^School of Public Leadership, Stellenbosch University, Stellenbosch, South Africa

**Keywords:** corruption, healthcare, legal profession, medical negligence, State Attorney's Office

## Abstract

The article is an empirical attempt to research, analyse, and dissect the corrupt involvement of legal practitioners in illegal and fraudulent acts, and mainly their involvement in litigation associated with issues of medical negligence. This is done primarily but not exclusively through the utilisation of several qualitative research methods, including the content analysis of primary literary sources such as official state documents, existing legislation and court proceedings and personal interviews with senior public servants, as well as secondary sources. Beginning with a short exploration of South Africa's public legal terrain and the fears of sections of the statutory leadership of the legal profession, the article continues with the identification of key findings in several the country's provinces, the modus operandi of the corrupt individuals and groups, as well as the monetary, financial and social repercussions of such actions.

## Introduction

The 2030 Agenda for Sustainable Development adopted in 2015 by the United Nations' General Assembly requires states to reduce bribery and corruption in “all their forms” by 2030. Analysis has shown that from the outset the UN has espoused what could be regarded as a human rights-based approach to corruption, which as noted by Cecily Rose has its limitations [(1), p. 405].

Internationally, there is a widely accepted position and belief that corruption is one of the most important impediments of human development and the promotion of human rights in general, especially for the poor and marginalised masses in Africa and globally. South Africa, of course, is no exception to the rule as a wide variety of corruption perpetrators throughout the years both in the public and private sectors have taken advantage of lack of accountability, honesty and transparency and especially the impunity of the corrupt individuals, companies, groups, mediators, relatives, families, and factions.

The whole world celebrated the peaceful transition from apartheid to democracy in South Africa with Nelson Mandela's African National Congress (ANC) becoming the first democratic government in the country. The new path begun with a short period of peace, unity of purpose and the unsuccessful planning and implementation of the first socio-economic plan, the Reconstruction and Development Plan (RDP). The policy was abandoned after a year and a half and was followed by the Growth, Employment and Redistribution (GEAR) Plan in 1996 (2, 3).

Following these “strategic changes” of the Mandela and Thabo Mbeki led governments, the first massive corruption case took place under the latter's cabinet. Named the “Strategic Defence Package” also popularly known as the “Arms Deal” its cost was calculated at the time at 30 billion South African rands (equivalent to UDS $ 4.8 billion). Despite the existing strong evidence presented publicly against the then Vice President and later President Jacob Zuma at the time, it is only at present that the senior politician will appear in Court as Accused number 1 in June 2021. In the meantime, recent economic/financial research has shown that the cost of the arms deal is calculated at 142 billion rands in today's value (4).

As things stand in South Africa at present, most social, political, and economic analysts have agreed that the financial and political damage caused by the “state capture” period during the Jacob Zuma regime (2000–2018) made the “Arms Deal” look like a “drop in the ocean” at many levels.

As (5) mentioned in their analysis of state capture several individuals and their companies shaped government compositions, influenced the structures of public departments, laws and regulations to their own advantage. In such cases government officials at both political and administrative levels are provided with illicit private gains. In such processes anti-corruption laws, regulations and agencies are “blocked.” As the process unfolds, the trap of the captured economy becomes a “vicious cycle” in which policy is completely undermined by collusion between dominating companies, groups or families, and state politicians and administrators who accumulate corruption-ridden wealth to the detriment of their citizens, especially the poor and the marginalised.

In South Africa, the main but not the sole, representatives of the “private sector influencers/capturer” group consisted of an Indian businesspeople family, the Guptas, who immigrated to South Africa in the early 1990's and begun what was to become a widely diversified conglomerate with Sahara Computers, an information technology company distributor as the start. The establishment of the Oakbay Investments, a holding company, comprised of media, engineering and mining and its continuous diversification of activities became a complexity of note, a foundation of a multifaceted reality of state capture. All of the roads to the path of corruption and multi-million and billion business initiatives were rooted on personal, family, political, economic and financial chicanery (5–11).

The country's political leadership at all layers of the state apparatus (national provincial and municipal) during the Zuma presidency had a complete domination of all key state institutions, departments and agencies including the tax authority (South African Revenue Service), the anti-corruption institutions such as the National Prosecuting Authority, the police as well the most important structures, functions, and processes of the ruling party (the African National Congress). Throughout the country, the ruling party's machinery through its political and administrative deploys controlled job allocation, the supply chain and procurement machineries and all administrative set-ups. On occasions the utilisation of “mediators” became compulsory as corruption drivers. Such realities pointed to the fact that state corruption had been transformed into state capture that had been institutionalised throughout the country where private individual interests had become the state's modus operandi. Private financial and material gains jettisoned the peoples' interests and needs that worsened by the day due to individuals' and groups' greed and avarice. As the process unfolded however, a wide variety of societal and political players such as non-governmental and civil society organisations, trade unions, printed and social media, religious leaders and the opposition political parties led a serious pressure for the government. The unfolding reality became more concretised by the then Public Protector's report titled State of Capture which was released October 2016 (12).

The document highlighted vividly the existing patronage network led by the Zuma-Gupta alliance used state departments, entities, institutions and companies to enrich themselves and those close to them. The structure and process cementing such relationships has been also labelled “radical economic empowerment” denoting the control of the heights of the country's economy by the previously disadvantaged Black population.

Following the recommendations of the Public Protector in 2016 the then President Jacob Zuma established the Zondo Commission of Inquiry in January 2018 aimed at an investigation allegation of fraud, state capture and other allegations in the public sector including organs of state. Until today the Commission has interviewed over 300 witnesses including politicians, state administrators, businesspeople, including Jacob Zuma and several the country's previous Ministers (10).

In one of the latest appearances in the Zondo Commission a senior researcher of the highly respected international non-governmental organisation Shadow World Investigations testified that the amount of R49,157,323,233.68 rands was paid by the South African state on contracts included in State Capture processes (13).

Throughout the world, including South Africa, the common denominator characterising a legal person in the private or public service is the expectation that he or she will be honest, reliable and accountable, in other words, a person of integrity. These ethical and professional virtues begin at home, are inculcated throughout basic education and it is hoped, will be entrenched at the various Law Schools which ought to not only produce knowledgeable attorneys, advocates, magistrates or judges, but also ethical persons. These persons of the highest moral calibre are not mentored only in the various professional Codes of Legal Ethics, but also into the firm adherence to the practise of integrity and honesty at all levels of life. The intimate relationship of moral compass and legal ethics is the foundation of legal practise, the roots of a strong will to avoid temptations, pitfalls and traps.

As any legal person worth his/her degree knows, health is a fundamental human right, especially for the vast majority of people. Every decision in one's life can be considered difficult, but some are especially so for a legal person who has to take a stand and make a decision of what is right and what is wrong. Conflicts of belief can lead to either honesty or corruption. This is the case for issues of health too.

In South Africa, a country blessed with diverse cultures, customs and traditions, healthcare is definitely a matter of life or death. Decisions made have direct and indirect effects on the health and lives of people. The choice of a moral or corrupt action on the part of private or state legal people has a direct or indirect effect on the lives and health of millions of people. This is the central theme of this article.

## The South African Public Legal Terrain

There are similarities and differences between private legal practitioners and those working at the State Attorney's Office. In fact, there are three main categories of legal practitioners working for the state and state institutions. State attorneys are responsible for dealing with a wide variety of legal issues of businesses, individuals and corporations in key fields including taxation issues, corporate and business law, litigation of a criminal and/or civil nature, estate planning and property issues. In their field of duty, they represent state institutions and departments in the legal processes and transactions against or for the State. Their work, functions and processes do not differ from those of other attorneys operating in the private sector.

However, state attorneys deal with a number of issues that are specific and could be classified as “specialized” and demanding “special skills,” such as appearing in Constitutional Court cases and complicated probable corruption and fraud cases and instructing junior and senior advocates in complicated cases.

The Attorneys Act 53 of 1979 (14) is very specific on the requirements, steps, processes and regulations to be followed before the appointment of such personnel, who must display honesty and fairness, and whose qualifications include specialised training, followed by the Attorneys Board Examination, that operates under the auspices of the Law Society. Following the candidate's admission as an attorney and practise of between 2 and 3 years, the admission to the state institution can occur.

State advocates operate as “public prosecutors” in the country's High Courts and are appointed by the NPA National Prosecuting Authority and the Office of the National Director of Public Prosecutions. The position requires an LLB degree and registration on the “roll” of advocates. The process is completed once the Court is satisfied that the candidate is able and qualified to join the group and the profession in general. A pupillage is a compulsory step to be followed in the process. It is a 1-year apprenticeship that includes a final examination (the National Bar Examination of the General Council of the Bar), a pre-requisite to join the Bar. A pupil advocate is paired with an experienced advocate during the period of the pupillage, in order to be guided through the dynamics, processes, systems and organisational details of the professional “chambers” and the courts. It is an unpaid learning process.

The admission to the representative body of the profession (“the Bar”) signifies that the successful applicant can practise as an advocate in the country. The local chapters of Societies of Advocates of the General Council of the Bar of South Africa provide the compulsory training. Once the admission to the “Bar” has been achieved, a direct application for a State Advocate position can be placed with the National Prosecuting Authority.

Experience and expertise are deemed important for the position. Public prosecutors are regarded as the country's criminal law “gate-keepers” and are the state's representatives in the prosecution of cases. They are the decision-makers in prosecuting criminal cases that have been investigated and decided upon, based on existing evidence by the relevant state authorities (15).

Over the last few years, there has been a realisation that there is an ethical crisis in South Africa's legal profession, although some will deny it. At an official platform for Law educators, the representative of the Law Society in the country revealed that “thieving attorneys” peaked R200 million in 2014. It was stated that one of the country's Law Societies received 6 000 complaints because of the members' “I don't care attitude.”

The Chairperson of the country's Law Society said that the profession had ceased to be the guardian of integrity and dignity for the customers who expected to be served by a “fit and proper person.” He provided a number of cases where legal firms bribed others, treated poor people with disdain, and operated without diligence or care. This meant that the Legal Practises Act that guides the professionals to be ethical, honest and fair to all was violated. It was emphasised that legal practitioners were not “hired guns” but the “implementers of the country's Constitution.” Their ultimate role was to administer justice and to perform their duties with the highest degree of dignity and integrity. The Chairperson surmised that the profession should be the beacon for justice, not a life of luxury (16).

Throughout the years, there have been reports that the legal profession in South Africa has faced corruption risks in a variety of levels in their relations with clients, state institutions and individual, businesspeople and the private sector in general. Such realities have led to reports associating legal practises with briberies, acting as mediators in corrupt private and public businesses, consulting in corrupt schemes, associated with illicit payments, invoicing and billing fraudulent acts (17).

It is within the above context that the findings of the South Africa's SIU (Special Investigating Unit) pinpointed in their investigation for the collusion of three senior advocates with the Office of the State Attorney. This was classified as a “tip in the iceberg” as the existence of massive fraud and rampant collusion between private legal practitioners and the Office of the State Attorney has been uncovered on several occasions. While a Disciplinary Committee of the State Attorney was in process, he resigned while the criminal investigation of the three advocates was in process. About 26.5 million rands were involved in the cases, some of which was recovered through the Special Tribunal's civil process. One of the three advocates offered to repay some of the irregular amounts. It was said that this was just the tip of the iceberg in a vast SIU investigation into matters relating to the Office of the State Attorney (18).

It has been noted that law practitioners' corruption risks and acts have been reported mainly through connexions, relations, and actions related to politicians and government officials as well as other law firms, in terms of brokerage, consultancy and counselling, accountants and other law firms (17, 19).

These facts become a reality throughout the spectrum of the legal profession despite the existence of laws such as the Prevention of Organised Crime Act 121 of 1998, which provide the anti-corruption state authorities with the ammunition to freeze and ultimately seize the proceeds of crime (20); the Protected Disclosures Act 26 of 2000, that protects citizens from discrimination in cases where they blow the whistle on corruption (21); the Financial Intelligence Centre Act 38 of 2001, is a law against money laundering and imposes an obligation on lawyers, estate agents and brokers to report suspicious transactions (22) and the Prevention and Combating of Corrupt Activities Act 12 of 2004, considered the ‘major anti-corruption initiative' in the country (23).

Corruption in the health sector *per se* has been on the microscope for many years throughout the word as it is a phenomenon that affects life, infant mortality and especially children mortality rate negatively. As the pioneer work of Vian [(24), p. 87] pinpoints corruption in the health sector in society is directly related to lower levels of health expenditure in the GDP (gross domestic product), and poor health outcomes. The wide variety of corrupt acts in the sector includes, bribes, theft, political, and administrative fraud and misinformation, procurement and supply chain structures and processes medicine distribution, low quality services as well as interactions amongst health professionals and patients, and hospitals and suppliers [(25), p. 226–227].

International research has pinpointed conclusively that corruption in both the private and the public health sectors is deadly. It has been shown that in many underdeveloped countries, over 80% of the population experience corrupt practises in the health sector. In developed countries with flourishing private and public sector health structures and processes corruption take a wide variety of forms such as exorbitant prices, medical schemes and hospital overbilling [(26), p. 529].

Inevitably, corruption in the health sector takes a wide variety of forms and the corruption culprits in the empirical literature include administrators, professionals, salespeople, politicians, medical doctors, and procurement administrators (27). Rispel et al. (28) showed the corruption levels attached to provincial departments of health irregular, unauthorised, irregular, wasteful, and fruitless expenditure. These official realities have been described as the foundations of corrupt behaviour. Health care corruption has negative effects both on patient care and recovery as well as the morale of honest healthcare workers. Earlier research in South Africa as back as 2009 has shown that despite the fact that the poor and outdated conditions in the public health sector could be the foundation of the vulnerability leading to corruption, the situation in the private sector was not really different at a number of levels [(29, 30), p. 1353].

The official and thoroughly researched findings of the Auditor General South Africa for 2017 on South Africa's national and provincial Health Departments pinpointed as the roots of corruption and fraud the following realities: lack of appropriate political oversight in the sector and especially the relationships of state institutions and the elements of the private sector; lack of existence or/and coordination of anti-corruption policies and agencies; nepotism and conflict/s of interest; blockages taking place due to complicated policies; inappropriate or inadequate compliance systems; governance systems with limited functional institutional capacity; inept personal competencies in relation to governance, policy planning and implementation; role-players operating within and across institutions; serious fraud, collusion and corruption amongst senior politicians and/or administrators in most instances and state-led health services organisational lack of efficiencies, as well as lack of financial and risk management systems [(31), p. 57–58].

In many ways it can be stated at the outset, that healthcare is only one of the terrains where legal professionals are involved in unethical practises, as research has concretely shown (32–35).

All these cases are isolated and despite the fact that they appeared in press reports, the realities of the expansion and serious importance for serious and comprehensive empirical research cannot be underestimated. Such research is fundamental in the effort to fill a major gap in the existing literature on the topic covered in the article. It is an effort that points amongst others to the prioritisation of decisive and well-organised anti-corruption measures against a multi-billion of rands reality. Such research describes the realities and repercussions of what can be called a “health care capture of a different kind” this time from individuals, groups or syndicates with a wide variety of legal, para-legal undergraduate or post-graduate degrees or diplomas. It is an empirical effort rooted on a wide range of primary and secondary sources including face to face interviews with first-hand knowledge of the realities of a situation no law, regulation or rule can stop, at least now.

## Methods and Data

The methodology framework utilised in this study was qualitative, with scrutiny of primary and secondary sources, such as official state and court documents and reports. The case study was rooted on the interpretative perspective and based principally but not exclusively on the deep knowledge of a variety of government officials with direct involvement in the existing situation.

The purposive sample utilised (a key primary source) (Interviewee 1; Interviewee 2; Interviewee 3; Interviewee 4; Interviewee 5; Interviewee 6; Interviewee 7) was based on the researchers' knowledge of seven senior management personnel in national and provincial Health Departments who have first -hand information of key issues associated with the realities of the relationships of the state institutions with the wide variety of legal challenges and problems facing the existing healthcare landscape in South Africa (36). The number of interviewees has been in line with the works of Dworkin [(37), p. 1319–1320] and Charmaz [(38), p. 113] who have advocated smaller number of interviewees in such a qualitative based research.

Data was collected through face-to face unstructured phenomenological interviews in Johannesburg and Durban and the interviewees shared their everyday, first-hand experiences with the researcher as Bryman and Bell [(39), p. 4] have described. In these interviews, the problems and challenges facing civil servants in the healthcare sector faced in their everyday and medium- and long-term duties and responsibilities in relation to the representatives of the legal profession at all levels. Their anonymity and confidentiality were guaranteed.

The recorded interviews were transcribed verbatim and the use of the NVivo Version 10 software was utilised in the facilitation of the organisation and analysis of data. In the process the qualitative inquiry was transformed beyond the codification and retrieval of data. It integrated the qualitative linking with modelling, shaping coding, and modelling the data as Wong [(40), p. 15–16] has shown.

The utilisation of primary and secondary sources were based on the nature and particularise of the project's aims and objectives.

Hence the decisions on the utilisation of primary source was based on the researchers' strong belief that the need of original materials is crucial as one key foundation of the research. This because a primary source of information is true and original; it cannot be evaluated or interpreted. In the fields of social sciences and humanities these sources are the true, first-hand and direct evidence of people, objects, institutions, events, or actions, scientific article presenting original findings and producing statistics, historical documents such as pamphlets, letters, pamphlets, ideological manifestos, political tracts, photographs, video footages, interviews and transcripts, eyewitness accounts, autobiographies, social media entries (Facebooks, tweets, Instagram, electronic mail) (41).

The primary sources utilised by the researchers included State Law Enforcement Documents (42); government institutions and departments official reports (12, 43–45), South African laws associated with the article (14, 20–23) and Parliamentary debates and state Commissions of Inquiry (10, 13, 46, 47).

In the utilisation of the secondary sources the researchers were provided with guidance, confirmation, interpretation, new knowledge, commentary, interpretation and occasionally analysis of existing primary sources. On occasions such sources located primary sources in their real context. This is the cases on many occasions as these secondary sources are in most cases produced after events that have taken place art a specific time. Hence the provision of such a historical context could be critical in understanding of events or phenomena. Newspaper and scholarly journal articles without new serious contribution to knowledge, monographs written on a specific issue, newsletters of organisations and biographies are amongst others important secondary sources for research.

In the triangulation process the researcher utilised printed and digital secondary sources such as academic articles (2, 4, 5, 7, 11, 17, 25–27, 37) and newspapers (16, 18, 34, 48–62).

The analysis of these sources was based on a thorough content analysis of these print and electronic media, thus taking advantage of the triangulation of research techniques.

Within this context, the research attempted to utilise an already existing knowledge of the healthcare industry and its particularities, which was used in order to widen the scope of corruption knowledge in a new empirical territory. The research findings of the study are trustworthy and credible because the research was conducted according to the principles of good practise and the interview transcripts and research findings were submitted to all informants to confirm their authenticity.

## The Corrupt Relationship Between the Legal Profession and Healthcare and their Repercussions

### Introduction

The present section that identifies, analyses and dissects the corrupt relationships between the legal profession, healthcare in the public sector and their repercussions is a serious and well-researched contribution to the understanding of the realities and dimensions of key role players in the processes of planning and implementing corrupt acts and behaviours and the role of key state institutions. The reasons behind acts that are presented and analysed for the first time are the root of the creation of new knowledge.

Having established the existence and deep roots of the legal profession's appetite in defrauding and taking advantage of weaknesses and challenges facing public healthcare, the political leadership of the National Health Department organised a medical/legal 2-day Summit to address the issue.

Government, civil society, all provincial MECs and their key administrators, all professional bodies in the healthcare terrain as well a large number of health professionals, researchers and academics, legal fraternity practitioners at all levels and representatives from the World Health Organisation, listened attentively to the National Minister of Health, especially when in his opening keynote address he indicated that the “lawsuit crisis in South Africa” had reached very similar levels to the one in Australia that ultimately led to the total collapse of the country's health system 15 years ago, and the turmoil in the USA with systemic crises in the 1970s and 1980s.

The South African crisis, he said, was like an “explosion” in medical malpractice litigation that has led to a skyrocketing of claims but does not seem to be guided by known trends in malpractice and medical negligence. He mentioned that the crisis that South Africans are faced with is not a crisis of public healthcare, but one faced by everybody in the health profession, both in the private and the public health spheres (51).

These pronouncements could come across as mere dramatic calls but a scrutiny of several reports during the past few years make such statements very believable and accurate (48, 51, 52, 56–58, 63–72). While this article will demonstrate the direct and indirect involvement of private legal firms and State attorneys in unethical acts that have been taking place for many years throughout South Africa's healthcare terrain, there is no more concrete evidence of the phenomenon and its dimensions than the Gauteng Department of Health.

### The Analysis of Data

#### The Political-Legal “Alliance” in the Gauteng Province

While it has been widely reported that the Gauteng Health Department has faced a severe structural and financial crisis over the last 10 years, the serious case involving the then MEC of the province, Qedani Mahlangu and her favourite law firm Ngcebetsha Madlanga Attorneys, only attracted limited publicity even though hundreds of millions of rands were involved. It surfaced that the firm was paid R162m for “services rendered” to the Departments she headed: R103m by the Gauteng Health Department and R59m for work done for the Department of Infrastructure Development (DID), which she previously headed in the 2012–2014 period.

Jacob Mamabolo, the present DID MEC, indicated that the firm was appointed in late 2012 as part of a Panel of Attorneys' tender. There was no quotation and the firm was paid for actual work done, which “was billed on a time and cost basis” (ref). Such action is against the dictates of the Public Finance Management Act (PFMA). The firm's work in the Health Department included legal advice and the authoring of Service Level Agreements (SLAs), three disciplinary hearings (costing R2m, R1.7m and R1.1m each), a litigation audit (R7.3m) and legal advices on a single forensic report (52, 53). It was interesting to note that the law firm acted as Mahlangu's personal lawyers before the call for her to testify at the Esidimeni Arbitration Hearings chaired by Judge Moseneke. In the process, it surfaced that the two Departments she led were the only ones in the province that had offered work to them (52, 53).

#### The Life Esidimeni Tragedy

The MEC resigned in February 2017 following her humiliation in the Life Esidimeni tragedy's hearings. At least 144 psychiatric patients died after they were transferred to institutions and non-governmental organisations that were not equipped to look after them. It has been announced officially by the new Gauteng Health Department MEC that all the firm's bills are under scrutiny and undergoing a professional assessment by the provincial Law Society (73).

These acts took place during a period of perpetual crisis at the Department, beset by the deepening demands for unpaid claims by a wide variety of service providers, the attachment of bank accounts and paralysed services throughout the operational spectrum (74).

The Life Esidimeni tragedy was the worst of all, but the turmoil and chaos experienced at all professional/organisational levels in the Health Department were perpetual. There were continuous withdrawals from hospitals of nurses because the service provider was unpaid for months; no payments were made to NHLS (the National Health Laboratory Services) that provides illness diagnoses through testing (calculated at R2.58b); the Health Department had all its furniture and computers attached by the Sheriff for failing to pay a medical negligence pay; 14 of the Department's bank accounts were attached for a R33.7m claim; its head office phone lines were cut by TELKOM; they had to deal with over 2,000 medical negligence cases simultaneously; they were obligated to pay for all expenses for the Esidimeni hearings and arbitration and they are now facing the monetary pay-outs to the Esidimeni victims' families. This was at a time when times became tougher, due to increasing unemployment, poverty, retrenchments, the higher cost of living especially after the VAT increases, higher medical inflation, and population increases with new masses of people becoming dependent on public healthcare (75, 76).

#### An Anti-corruption Investigation: A 9-Year Conundrum

In the meanwhile, the findings of a 2008 Hawks investigation into the province's finances that began in the 2008/2009 period were finalised only in March 2017, and a report was prepared. It pointed fingers at Brian Hlongwa, the former Gauteng Health MEC (at present the ANC's Chief Whip in the Province) and several people in “closed circle.” The Hawks have been given new instructions to continue the investigations and there is a “strong push” to charge the provincial leader, given the release of the report that has been described as “damning.” In terms of Director General Hlongwa's corruption allegations, according to the report, the damage between 2006 and 2009 is estimated at R1.2b, and misspending had led to unaccounted funds of over R7b over the period of 5 years (53, 77).

Following the awarding of a R1.2m pay-out for each of the 135 claimants directly affected by the Life Esidimeni tragedy, the Gauteng Health Department needed to deal with one of the most common, murkiest, multi-dimensional, collaborative and yet interesting, legal cases planned, which was led and manipulated by legal syndicates at all levels and positions within the market and state institutions. The Gauteng government leadership announced at the time that there was commitment on their part to allocate the money to the relatives of the deceased through the Office of the Premier, although their source was not known because they were not included in the financial year's health budget (78).

#### The General Picture of Legal Demands, Mediators, and Negligence

Every year, more than 23 million people have utilised the provincial Health Department's hospitals, clinics, programs and ambulance services and health programmes. By the end of the 2017 financial year, the Gauteng government had to deal with more than 2,317 cases for “medical negligence” against the Health Department. The “contingency liability” stood at between R18 to R21b (43).

In almost all cases, these legal actions against state institutions have been initiated by members of the legal profession who over the years have taken advantages of circumstances to enrich themselves illegally and immorally. In most instances, they collaborate directly or indirectly with medical or nursing staffs who direct them to existing health problems facing patients.

There have been cases where lawyers themselves or their “representatives” (medical practitioners, nurses or “mediators”) are “searching/haunting” public hospitals mainly to discover patients with medical problems, usually children with defects. After making their own notes or illegally obtaining copies or originals of medical reports, they file cases against the Health Department (79).

It has been reported that since the beginning of the 2017/18 financial year, that the Gauteng Health Department had paid out over R400m for surgical negligence, neo-natal and maternal medical negligence. Most of these cases (over 50%) are for cerebral palsy (43).

It has been said, in addition, that there were plans to deal with these challenges directly during the 2018 financial year, with a number of attempts on the part of the leadership to educate middle managers on how to militate against issues of medical negligence through staff workshops with clinic and hospitals personnel, including state attorneys, board and council members. In a number of these workshops, hospital and clinics managers were to participate in the effort to solve incidents of negligence and corrupt practises (75).

Inevitably, such realities have led to negative effects on the Department staff morale, as most of them have witnessed the relevant authorities and anti-corruption agencies confiscating and attaching their computers, photocopy machines, laptops, chairs and tables. While these acts continue, the Department and its legal office continue the search for the corrupt “legal eagles” and their collaborators who are “preying” on weaknesses and gaps in the existing stems to loot the provincial healthcare budgets. Again inevitably, the existing situation is not sustainable as the cycle of corruption continues (78).

#### Legal Collusion: The Office of the State Attorney and the Legal Terrain

While attorneys and their collaborators in the provinces have over the years taken advantage of existing human resources and organisational weaknesses of the Department's systems and processes, the recent exposure of the direct and indirect involvement of legal persons in the Office of the State Attorney in collusion irregularities with private lawyers in the looting of the state coffers and budgets, provides a new direction to the study and fight against corruption.

The Office's key task is to provide honest, fair, well-researched and informed, accountable and transparent legal advice to all departments of the national and provincial governments. A senior Department of Health administrator with many years of experience queried the role of the state attorneys by pinpointing the fact that for a long time, it was known that state attorneys did not file court papers in time, did not attend seriously to litigation matters against the Department, colluded with private lawyers and mediators, and were instrumental in settling out of court, exorbitant financial demands, even when they themselves had defended such cases as their position required. It was felt that the existing “syndicates” operated either separately or on occasion, in collaboration. This collusion was made easy by the existing mismanagement and maladministration of the national and provincial offices. There were several prospective whistle-blowers who kept quiet for years on such issues of importance (80).

There have also been reports pinpointing the shedding of light on several collaborative corrupt operations already in existence due to the collusion of private law firms with State attorneys in a number of elaborate scams that defraud health and other departments purposely, through state attorneys strategically losing cases or settling out of court and sharing the pay-outs. The corrupt operations were based on what has been alleged to be collusions of State Attorney Office officials with private legal practitioners, who collaborated in utilising fictitious or real litigants to defraud the state through several existing avenues. The recent investigative team appointed through a presidential proclamation led by the Special Investigation Unit (SIU), has been instructed to scrutinise whether the Office of the State Attorney and/or members of its staff have: violated the dictates of Prevention and Combating of Corrupt Activities Act, 2004; committed unlawful or improper conduct that has led or could cause serious harm to the interests of the South African people; indulged in unlawful expenditure or/and appropriation of public property or funds, and participated in irregular, unlawful, unapproved acquisitive acts or transactions, that have a bearing on state property, and/or negligent or intentional loss of public money or damage to public property (81).

#### The Minister of Health and the Spreading of Corruption

On several occasions, the country's Minister of Health has provided several examples of fraudulent medical negligence claims, including that of a 19-year-old, whose lawyer claimed R25m for his client suffering from cerebral palsy when he was not, and a claim of R70m for a botched circumcision that never took place. Many such claims in Mpumalanga, Limpopo and the Eastern Cape were also withdrawn after serious investigations by officials of the Health Departments and the SIU. The Minister indicated that in the second case, the Department's legal team dealt with the State Attorney's Office and there was no cooperation, while the provincial MEC strangely knew nothing about the case. When the file was examined by an expert of a state hospital, it was discovered that no circumcision had taken place. Instead, the claimant was seriously ill, and his life was saved by the hospital. It has been estimated that such corrupt acts have cost the state over R80b and have cast a shadow of doubt on all aspects of corruption. This because there is strong evidence of direct involvement of state and private lawyers as well as collaborators on the medical and mediating stream operating (82, 83).

The networks to be investigated have been over the years involved in hundreds of cases that are lost or settled out of court without proper scrutiny of the facts and details, and involve a number of networks, sections, offices, groups and individuals. As a further example, there is already an investigation in terms of why 80% of the health department's claims in one of the regions in the Eastern Cape, are done by only 5 attorneys. Once the SIU investigators visited an office of one of the five firms, the number of existing cases was immediately dropped (59). Given the fact that such an investigation is destined to cover all existing State Attorney Offices in South Africa as well the complexity of the issue, the 1 year envisaged for it to draw its conclusions seems very short (81).

There are recent reports indicating that the investigators have already opened a number of criminal cases against private law firms as well as state attorneys who have “bungled claims against the state” intentionally and shared the pay-outs in the form of kickbacks. Most of these in the case of state attorneys have been found in the Eastern Cape, where the settling of cases out of court is the highest in the country, at least at this juncture. The SIU investigators have also confirmed that throughout the country, health claims are the most vulnerable in terms of scams and collusion of this kind. It has been reported that an Eastern Cape private law firm has initiated 28 cases against the Health Department for a total amount of R442.4m [each one for R15.8m (55)].

The Eastern Cape has been also mentioned as the province that has faced the greed of lawyers who have perpetually milked the Health Department with a multiplicity of bogus and fraudulent medical claims, that had pushed its legal bill to over R17b or 72% of its total budget that stands at R23.6m (84). Of this amount, R900m has been claimed by three attorneys in the province. In a paid claim worth R10m, the claimant herself received R100 000, with the rest going to the lawyer (85).

#### The Collaborators and “Mediators”

There are public servants within the Health Department who help lawyers in state fraud by photocopying documents that are instrumental in the duplication of claims. “Spotters” who are used in hospitals and clinics are those stealing patients' documents, selling them to lawyers who then institute claims. In a number of cases, the claimants themselves have no knowledge of the claims in their own name. It took at least 3 years of such acts to convince the Department of Health to employ two private legal firms as investigators of the claims' authenticity. When local firms that have claimed realised this, they immediately withdrew cases worth R75m, while over 30 medical facilities were identified as “soft targets,” mainly in the former Transkei, following the legal investigations, the private legal firms targeted the East London area. The Eastern Cape Health Department, however, had identified several culprits, especially those who claimed damages without the patients' knowledge. Such cases were organised by “spotters” who concentrated on maternity cases, accidents and orthopaedic operations (85).

The KwaZulu-Natal Health Department leadership, in contrast, which received the second highest number of medical negligence claims following Gauteng, told the Joint Standing Committee of Appropriations and the Portfolio Committee on Health, that the province's contingent liability for medical litigation stood at R10b (47). The 2016-2017 expenditure for claims was R243m, which was not included in the provincial budget (44).

It has been confirmed that a significant number of private legal firms advertised their services in hospitals through pamphlets and mediators, through mostly medical staff and nurses, whose “job” is to convince those with medical problems to choose specific attorneys. There have been many cases where medical practitioners in public hospitals colluded with law firms by handing them patient files. Attorney's “helpers” have also been visiting early childhood centres to find children with cerebral palsy or other birth complications. The Northern Cape had recorded R1.2b in pending legal claims, an increase of over 100% in comparison with the previous year. In the Limpopo province, the pending medical claims increased by 133%. They recorded an average of R30m per year from 2013 to 2015, while they stood at R70m in 2016–2017 (47).

Such realities indicate clearly that the corruption cycle cannot consist of only two parts (private law firms and state attorneys). There are more parties involved. This is even though it is considered “easy” for a state attorney to lose a case deliberately. The key problem facing the authorities is that there have been cases where close collaboration amongst different parties is evident as malpractice dominates at different levels and departments (80).

Other reports indicate that a list of “kingpins” has been in the hands and surveillance of the SIU, as there have been cases of officials who earn R200 000 or less a year, but have been driving very expensive vehicles, while a number of the key private legal firm suspects had initially resisted providing information but have changed their minds and are prepared to participate and do so (59).

As early as 2016, the Public Service Commission report on the State Attorney's Offices had showed that its members had lost 70% of its cases (86). One could explain such figures through a multiplicity of reasons, the most appropriate being inefficiency and collusion.

Lack of deliberate plundering of the state's cases could also relate to the fact that specific departments are ignorant of a specific case as they have not been informed or even ignore existing information provided to them (79).

#### The Legal Fraternity Responses

Despite the strong and pertinent statements of its President cited above and the empirical realities identified in the context of the present article, a senior leader of the Law Society of South Africa (the joint President of the Society), accused the state ministers who decided and implemented the Hawks investigation on private legal firms and State attorneys of being “opportunists,” by claiming that attorneys might have used illegal acts in order to defraud the government of R80b. This was' an attempt to tarnish the profession with no evidence' was the defence of the Society's leadership. It has been said that such allegations are harmful to the profession because it is perceived to be complicit to some corrupt activities. These “suggestions” have created a negative picture of the profession to the country's citizens. The Society accepted as fact that “some attorneys” are “unprofessional” and corrupt, but “sweeping statements without evidence and concrete complaints” are unacceptable. The leadership declared that they were “unsure” whether all lawyers operate lawfully and properly. There could be “maybe,” “somehow” “somewhere” “one or two instances” of unprofessional conduct, but these were sorted out through the actions of the regulatory body after investigation (60).

The “defence” of the body was in fact rooted on shaky foundations, as the reports of the Minister's meeting made it clear that there was no attempt of the three key ministerial speakers to denigrate or cast aspersions on all attorneys or the profession in general. By pointing out a number of concrete cases of collusion and corruption, the speakers identified concrete instances of collusion between attorneys and “collaborators.”

The R80b figure was most likely a combined figure for a period of years, based on calculations of direct and indirect malpractice and corruption at several operational, financial and monetary levels through the nine provinces, and the calculations could have been based on a number of official sources such as the provincial budgets, the Auditor General's reports, and the provincial Health departments' Annual or the Treasury reports. It has been accepted by all that most of this money is paid for cases of medical negligence, fought by law firms. The words of the legal society leader, especially the direct quotes that “there could be maybe, somehow, somewhere, one or two instances of corrupt lawyers,” have been completely proven wrong as the very society had stated that there were 33 attorneys struck from the roll of the Law Society of the Northern Province and 11 from the Cape Law Society in 2017 (60).

These numbers contradict the generalisations of the highest echelons of the Law Society of the country, but also raise questions about the investigating methods of regional law associations in the country and their position vis-à-vis the “floods of complaints” from health authorities and many citizens. While these associations can suspend members to investigate their professional integrity, they hardly pursue such processes. The regional legal associations have ceased to exist as from the 1st of November 2018, as the Legal Practise Act (87) dictates the existence of a national body only.

The 2016 Constitutional Court judgement in the case of Minister for the Executive Council for Health, Gauteng v Lushaba, acknowledged the fact that the provincial Health Departments faced a ‘flood of medical negligence litigation, creating a situation where, worst of all, is that “litigious lawyers seem to prosper and bureaucrats seem to get off scot-free” (42).

## Discussion

The complexity of realities, processes and relationships associated with the project's topic has begun with the fundamentals associated with the South Africa's public legal terrain and the expression of deep concern on the part of the elected leadership the country's legal profession.

The article continues with the identification of key findings in several the country's provinces, the modus operandi of the corrupt individuals and groups, as well as the monetary, financial and social repercussions of such actions.

The empirical evidence, the research findings and the analysis of data based on a wide and well-planned method has enriched the existing literature in both theoretical and empirical/methodological terrains. The new findings have most likely opened the path for new theoretical understanding of a complicated national, African and global phenomenon.

The diverse nature of legal corruption is evident from the direct participation of MECs of provincial Health Departments for many years leading to financial crises of their department due to senior politicians and administrators favouring particular legal firms against the dictates of key legislation against corruption such as the Public Finance Management Act (PFMA).

Such dishonourable relations led to deadly tragedies such as the Life Esidimeni tragedy where at least 144 psychiatric patients died that led to legal firms' bills while simultaneously undergoing a professional assessment by the Gauteng Province Law Society. Such realities point to the fact that several senior politicians had created a “closed circle” with legal firms. In the continuing process of the state's awarding of a R1.2m pay-out for each of the 135 claimants of the Life Esidimeni tragedy the article has shown that the provincial Health Department had to deal with fresh legal cases led and manipulated by legal syndicates. Such planned legal initiatives were in addition to more than 2317 cases for “medical negligence” against the Gauteng Health Department.

Such facts have been the creation of legal professionals who have taken advantage of gaps and alliances in to enrich themselves both immorally and illegally and immorally through their relationships with politicians, administrators as well as medical or nursing staff. Such collaborations and initiatives pale into insignificance when compared to the exposure of the direct and indirect involvement of legal persons in the Office of the State Attorney in collusion irregularities with private law firms in the looting of the state coffers and budgets. The confirmation of such a reality was the epitome of facts that have over the years surfaced through several reports exposing several collaborative corrupt operations already in existence. This was related to the collusion of private law firms with State attorneys in several elaborate scams that defraud health and other departments on purpose. These realities led to several criminal cases against private law firms as well as state attorneys who have “bungled claims against the state” intentionally and shared the pay-outs in the form of kickbacks.

Many research-based cases have shown conclusively that corrupt realities indicate clearly that the corruption cycle cannot consist of private law firms and state attorneys only, especially in relation to “medical negligence cases” facing a number of provincial Health Departments. The mere fact that records have shown that there were 33 attorneys struck from the roll of the Law Society of the Northern Province and 11 from the Cape Law Society in 2017 have shown that the attempt of the Law Society's to show that “there is an attempt to tarnish the profession with no evidence” was a complete negation of the existing reality.

## Policy Implications

The policy implications emanating from the empirical research are directly related to two wide terrains, the South African public service with special emphasis on the provincial and national Health Departments and the official representative of the country's legal fraternity, the Law Society, its sections, and branches.

It is widely acknowledged that the country's anti-corruption legislation and agencies are comprehensive and understandable, but there are several policies to be re-shaped and advanced at several levels.

The above need to be rooted on a clear understanding of the types and common areas and types of corruption, firstly within the micro context of a state organisation, agency or entity, or a private business firm (including a legal firm).

A process and analysis of existing situations will lead to a knowledge of parties involved directly and indirectly in corrupt acts and the scope for corrupt relationships, practises, and their causes. Such realities are directly related to the organisational environment which invites corruption.

This knowledge leads to planning and deterrence strategies and tactics through rules and regulations of “anti-corruption” behaviour rooted to ethics and the behaviour modification leading to practise of “good governance.” The latter is led by honest, decisive and servant leadership, management rooted on solid anti-corruption arrangements, solid experience and knowledge, rigorous accountability arrangements; planning and implementation of the best practise management systems; solid Financial Management, Budgetary control, costing, accounting, Asset Management, Internal Control, and Risk Management systems.

The final and key policy is related to the vulnerability of Procurement and SCM systems with special attention to prevention, deterrence, monitoring, analysing, detecting, investigating, and responding.

These are supplemented by the key role of the Internal Controls and Internal Audit and above all relentless employee awareness.

## Conclusions

There is no doubt that while no fraud or corruption can be tolerated as they are both direct violations of a basic human right such as health, undeniably it is the state's responsibility to fight against corrupt public servants as well as those who violate the laws of any country (including attorneys, “mediators,” medical practitioners or nurses).

All these categories of people, except for the mediators perhaps, have taken professional oaths to serve humanity and citizens with care, honesty, transparency, and accountability. On the contrary, in South Africa, and in many other countries, large numbers of them pursue power and money, not necessarily in that order. On many occasions, the corrupt in the public sector operate with disdain for their political leaders (who in turn neglect the responsibility of oversight). Arrogant disregard and recklessness for decisions made in the looting process are the order of the day. Evidently the actions of such unscrupulous public servants are shielded regularly from the expected repercussions of their corruption by protective politicians or their public or private institutions or professional associations.

The existence of legal and regulatory frameworks, mechanisms protecting the state and citizens against corruption and its repercussions, has not guaranteed that culprits and guilty parties face the consequences of their actions. This is due to several reasons including lack of accountability, crises in leadership, inept supervision, and political oversight.

There has been a general feeling amongst the country's citizen that the politicians and senior state administrators who rubber-stamped criminal laws such as the Prevention and Combatting of Corrupt Activities Act (23) and the Prevention of Organised Crime Act (20), and who deflect their responsibilities in fighting corruption and prosecuting the corrupt. In the process, they eventually lose the public trust (88–92).

In many ways, it has been shown that leaders both in the public and the private sector do not fulfil their ethical and professional duties diligently, despite the existence of political or professional oversight. Thus, the non-existence of the “accountability chain” leads to a wide array of bad decisions.

It is not easy for a senior state Minister to declare that corrupt public servants and other criminal elements whose activities threaten the citizens' healthcare must be arrested and that the public and private sector legal functionaries and their syndicates need to be investigated. Thousands of citizens joining public protests throughout the country demand the same, but they feel deeply that the public sector medical doctor who connives with members of the law profession believes that he/she is immune to arrest despite their deeds.

The arrests of the corrupt, however, if or when they take place, need to be followed by a well-planned and executed mission for the immediate recovery of the billions lost to avarice, greed, fraud, and corruption, followed by the immediate public exposure and prosecution of all involved.

In respect of the State Attorney's Office, there seems to be a feeling that a comprehensive review of the regulations, legislation and existing policy underlying the organisational, structural, systemic, and ethical foundations of the office, is desperately needed. This article provided evidence of corruption by collusion between the legal and healthcare sectors, pointing to systemic weaknesses and challenges facing the honest, efficient, and effective of the South African state legal services at different governmental layers. Enhancement of effectiveness, professionalism and efficiency mean nothing, unless they are founded on moral ethics.

The limitations of the study point to the fact that despite the empirical connexion of the “general/holistic” and the particular in a detailed and comparative mould the relations and realities studied and presented in the article need to be expanded not only to the whole country but throughout Africa. This is an important call to government anti-corruption agencies, non-governmental and civil society organisation, and university researchers to attempt the deep realities of corruption, collusion, market capture, alliances, relationships that exist amongst unethical individuals, groups and alliances that dominate markets, provinces, and whole countries.

## Data Availability Statement

The raw data supporting the conclusions of this article will be made available by the authors, without undue reservation.

## Ethics Statement

Ethical approval for this study and written informed consent from the participants of the study were not required in accordance with local legislation and national guidelines.

## Author Contributions

EM was the lead researcher and PP was his co-researcher. All authors contributed to the article and approved the submitted version.

## Conflict of Interest

The authors declare that the research was conducted in the absence of any commercial or financial relationships that could be construed as a potential conflict of interest.

## Publisher's Note

All claims expressed in this article are solely those of the authors and do not necessarily represent those of their affiliated organizations, or those of the publisher, the editors and the reviewers. Any product that may be evaluated in this article, or claim that may be made by its manufacturer, is not guaranteed or endorsed by the publisher.
