# Supplementary material for: Legal Profession and Corruption in Health Care: Some Reflective Realities in South Africa
Source: Front Public Health. 2021 Aug 25;9:688049. doi: 10.3389/fpubh.2021.688049 (PMC8424199; doi:10.3389/fpubh.2021.688049)
Supplement: Supplementary file 1 [file Data_Sheet_1.docx]

**TRANSCRIPTS**

**Interviewee 1, Senior Public Servant, Gauteng Health Department 17 October 2017.**

1. ***How the situation in the Department started with the legal firms that created all these problems in the process?***

Everyone knew and knows that the Department and the Infrastructure Development Department have been in financial trouble for years and this is because of the politicians in the province. Whatever happened in the departments went directly to lawyers and all these things were done without consultation and hundreds of millions of rands were involved.

In South Africa since 1994, whatever people say the truth is one, everyone wants to be rich and based on that each person, especially politicians and public servants make plans, the private sector people have planned all their lives. In the Department of Health there are thousands working, senior, middle and lower grades in the provincial officers, the hospitals, the clinics, everywhere. They have families, friends, connections etc. and they need to follow the laws of the country.

A lot of them, at all levels, have been trying and still try to achieve their dream of becoming rich meaning that they look for the opportunities to make extra money, possibly a lot of money. This means that when or if one of them is approached by a lawyer who also wants to make everyone and himself rich proposes something that seems easy or doable, a deal is done in most cases. It is a difficult decision that many make it because they can operate at a number of levels, operators, collaborators, mediators amongst other things.

Most corrupt lawyers are operators and know about all these rules, regulations, laws and they can guide the operators in the gaps that exist and could lead to money and a better life, and the middle of the road administrative clerk knows well the weaknesses, strengths and life of most politicians, including the senior ones. Most public servants in the administrative profession who understand the system will tell you that the lawyers and them pursue of business and money started from the first month after May 1994, the first elections of the new South Africa.

In the Department the situation started very soon and it continues for many reasons the most important is that politicians, most of them after money and power and they do not perform their services regarding their most important duty and responsibility, oversight. They love power and money and they operate with these things in mind. If you look around name how many are paying for their corrupt acts.

Nobody.

2.***Did the legal services passed through tenders? Were the tenders following the appropriate measures, laws rules and regulations?***

The Panel of Attorneys' tenders were without quotations most of the times, in my mind all the rules, regulations and the Public Finance Management Act (PFMA) were not followed that is why so many tens of millions went into a number of disciplinary committees, and forensic reports that lasted one or two days. Lawyers were paid for everything while the Department had its own administrative staff in these departments. The MECs one after the other messed up the situations and were never really accepted their and their lawyers’ responsibilities for the tragic death of over 140 Life Esidimeni poor people. The leaders were supposed to lead these poor people in a better life, but they led them to deal. the lawyers were there to guide them, give them advice, investigate the situation in the non-governmental organisations which supposed to accommodate the people. **The tenders were not done correctly and the situation that followed was expected and the law bills are still under investigation after so many months and suddenly the Law Society gets involved after all this time. To do what when everything has been destroyed.**

**Interviewee 2, Senior Public Servant, Gauteng Health Department 14 September 2018.**

1. ***How the situation in the Department started with the legal firms that created all these problems in the process?***

The situation started from the first day I started work here when I finished my Master’s degree 15 years ago. Those days’ things were a little bit better when compared with today. There were changes in the political leadership, but not really as many as at present, there were different circumstances politically. Then things started changing when President Zuma took over, because there were changes on the political situation, a number of new people took over the political situation in their hands. The road downhill begun, not that the situation was good before, but it became really uncontrolled. The politicians took over, re-structured sections and the Health Department went from bad to worse for the coming years especially when Mahlangu took it upon herself to ‘re-do’ the Health Department and her suborn attitude and greed led to major financial problems, a real crisis for many coming years. Suddenly a firm that few people have heard of, the Madlanga Attorneys, were contracted and then the situation of looting started and only finished after all these corrupt situations were exposed after years because Mahlangu was around for many years. The damage for the Health Department was R 103 million and R59m for work done for the Department of Infrastructure Development (DID) in two years as its Head. After that there was more.

1. ***Did the legal services pass through tenders? Were the tenders following the appropriate measures, laws rules and regulations?***

The situation in the Department knows that whatever has happened is possibly the result of the supply chain and procurement situation that has led to some realities that are known. The existence of the truth that the Department is real bankrupt, the perpetual demands for unpaid claims of most of the service providers, the attachment of bank accounts and paralysed services throughout the operational system, the realities that those lawyers close to the politicians are even paid before they finish the work.

Everyone in the Department is aware that the needs assessment for a lawyer of a legal firm is not done, jus the name crops up, there is no evidence of planning and budgeting or the definition of requirements according to the Treasury rules as well as the choice of procedure. All these realities mean that the choice of procedure pre –qualification Invitation to Tenders and Evaluations are simply pre-determined.

**Everybody knows that a solid Public Procurement system is there to fulfil its functions properly. When you feel well what I mentioned earlier certain crucial goals have not even planned, we understand that important principles are broken. This means that the goals that are the objectives that the PP system seeks to achieve are betrayed. This because the principles upon which the a code of conduct for all officials involved in the procurement process and are essential to the achievement of the PP goals is based are simply betrayed. So how do we expect to have integrity, value for money, equal opportunities, integrity and equal treatment for all providers.**

**When the agreements between the supply chain management people and their corrupt future or present ‘partners’ becomes a reality the utilisation of available funds, assets, and resources are lost to the Department, the province and above all the public interest. This reality means that corruption has won over honesty and integrity through direct or indirect collusion between government officials and bidders and or between bidders themselves.**

**These are realities that are happening as awarding contracts to a law firm could be on the basis of bribery, personal interests, political affiliation and ties such as kinship, family and business acquaintance.**

The situation has two faces because it consists of two elements, the integrity of the procurement process and the integrity of public procurement staff**.**  It is upon the staff to have the honesty and integrity to ensure that the whole public procurement is conducted in compliance with respective regulations and laws**.**This means that following the equal treatment of participants, ensures that procurement officials act in the public interest and administer public resources accordingly, guarantees accountability, and encourages public scrutiny.

The staff integrity that is crucial in public procurement especially when corruption is a serious endemic in a country in our case in most instances has been replaced by open or hidden violation of duties and responsibilities, punishable behaviour, waste or exploitation of resources, manipulating or abusing information, or corrupt activities such as collusive tendering or fraud.

**Interviewee 3, Senior Public Servant, Gauteng Health Department 18 October 2017.**

1. ***How the situation in the Department started with the legal firms that created all these problems in the process?***

Lawyers have been a major problem in the Department and other Departments in the province and the biggest tragedy they brought is the Life Esidimeni tragedy that has shaken the country and the world because it was the worst even in the history of health. This is not the only tragedy because if one looks at the situation in the department and other departments understands that there are major problems and challenges for many years and it seems they will continue to be there for years to come and these are here for a number of reasons. The lawyers have cost the department and other departments tens if not hundreds of millions for a lot of functions, a number of them that could be performed by administrators in the Department, but like everything else the decisions are made by the politicians and the provincial cabinets and administrators have no say. Whatever happened in the departments went directly to lawyers and all these things were done without consultation and hundreds of millions of rands were involved.

There was a serious mess in the hospitals because of the lawyers whose expenses had led to major problems because there was no money to pay service providers so the nurses left because they were not paid, the National Health Laboratory Services that has the experts in was bankrupt owing R2.58 billion. Because of the lawyers’ greed the department had all computers and furniture attached by the Court Sheriff and about 14 of the Department’s bank accounts were attached for a R33.7m claim and the telephone company (TELCOM) cut its head office phones because the department did not pay. Its head office phone lines were cut by TELKOM.

***2.Did the legal services passed through tenders? Were the tenders following the appropriate measures, laws rules and regulations?***

**If you look at the situation in the country and what is really happening in all provinces you will realise a number of things that are very clear. The laws, rules and regulations are clear how these legal appointments take place even when the provincial government and the department have very well-paid legal people and services and people who care ‘why these things happen’ these things happen because there are a lot of ways to avoid the directions and rules of the National and the Provincial Treasury**. How can you have a legally-based tender for law firms when you have over 2000 medical negligence cases simultaneously or when the Provincial Department, not the Provincial Government is obligated to pay for all expenses for the Esidimeni case**.**

The politicians, together with some administrators on occasions made the choices for the legal people because the cases continued to grow and the staff were even frightened to talk about these things because they were afraid**.** In all cases, most of which were lost no one knew why there were no tenders, they said tenders were there for ‘sass cases’, but even for them there were the ‘special legal advisers’ who were called ‘experts’, ‘senior advisers’, ‘senior advocates’, ‘senior arbitrators’**.** But if you asked the legal ‘brains’ in the Department they would not answer, they follow instructions.

Then the MEC would stand up and tell those she (and later he) trusted that Supply Chain and Procurement were really not needed badly because of the existing circumstances because the risk Management and the Internal Audit were really skilled. The people had no choice but to believe them and those close to them because they promised new staff incentives and key plans for all staff members so the situation would be soon changed for the better, they organised some seminars and talks in 2018 hoping to solve the problems that existed.

We heard there was a group of middle managers, in two sections that were trained by some advocate who was Senior Councillor in the Advocate’s Offices how to deal with medical negligence. There was also a plan to have similar workshops with hospital and clinic staff and the interesting part was that these groups would include state attorneys, and council and board members. This is strange because these groups supposed to know corruption, laws rules and regulations well and they ought to be capable of solving negligence and corrupt practices and acts at all levels.

**Interviewee 4, Senior Public Servant, Gauteng Health Department 19 October 2017.**

1. ***Did the legal services pass through tenders? Were the tenders following the appropriate measures, laws rules and regulations?***

The Panel of Attorneys' tenders were without quotations most of the times, in my mind all the rules, regulations and the Public Finance Management Act (PFMA) were not followed that is why so many tens of millions went into a number of disciplinary committees, and forensic reports that lasted one or two days. Lawyers were paid for everything while the Department had its own administrative staff in these departments.

The MECs one after the other messed up the situations and were never really accepted their and their lawyers’ responsibilities for the tragic death of over 140 Life Esidimeni poor people. The leaders were supposed to lead these poor people in a better life, but they led them to deal. the lawyers were there to guide them, give them advice, investigate the situation in the non-governmental organisations which supposed to accommodate the people. The tenders were not done correctly and the situation that followed was expected and the law bills are still under investigation after so many months and suddenly the Law Society gets involved after all this time. To do what when everything has been destroyed.

**Interviewee 5, Senior Public Servant, Gauteng Health Department 15 October 2017.**

1. ***How the situation in the Department started with the legal firms that created all these problems in the process?***

The situation has been happening as long as I remember in my career because lawyers are like corruption they have no colour or country and in most cases it has been evident in all terrains of life and the state there are no differences. Lawyers in almost all cases have to fight to get inside and make money in the private sector because in the private sector people who got jobs know that when they do wrong they are out and difficult to get new jobs, in the government sector things are easier for lawyers.

You do not need to be a brilliant legal brain to make easy money in the government sector and all these legal actions against state departments have become easy. This means that lawyers, independent or law firm’s initiative and activate cases in most cases taking advantage of connections and circumstances because we see them as immoral, corrupt and against the laws they supposed to defend.

They have for years collaborating with politicians, administrators, nurses, doctors in the hospitals, in many ways, directly, indirectly and in the middle. With these people they come in contact with patients who have small or big problems and then they sue the Department claiming hundreds of thousands or millions depending on the case.

The lawyers also find well- dressed ladies or guys as ‘’representatives’’ and collaborators use the services to serve them by finding new clients in the public hospital like Steve Biko and private hospitals where the rich and the middle class go. They find people who accompany patients with all these medical problems real or fake They find children with defects usually because they have better cases to win legally, the mediators /representatives have the opportunities to make the notes, go to the nurses and doctors and pass them to the lawyers, they are buying the copies and then the lawyers open the cases against the Health Department.

2.***Did the legal services passed through tenders? Were the tenders following the appropriate measures, laws rules and regulations?***

From what I know there are some cases where legal services pass through tender, but difficult to say how many or the reasons why this one goes and the others not.

To my mind this means that there is no equal opportunity and treatment for potential providers and this is against all laws, rules and regulations including the Constitution, it is a serious principle because the bid submission and the evaluation process are completely destroyed.

There is no fair treatment of legal and procedural fairness and from what we know when cases go through tenders the final decisions are pre-determined, so people ask ‘what’s the use?’’

The situation is that while it is expected that all providers will be treated equally and following the laws etc. throughout the procurement process, when it happens there are bidders who are given access to privileged information at some stage of the contracting process especially amongst layers, because they know the laws.

In the lawyers’ cases while a reasonable amount of time should be left between publication of the contract award decision and the signing of the contract to give an aggrieved competitor the opportunity to challenge the award decision, this never happens.

And these are only the tip of the iceberg.

**Interviewee 6, Senior Public Servant, National Health Department 14 September 2018.**

1. ***How the situation in the Department started with the legal firms that created all these problems in the process?***

There are many stories on this issue but I can only deal with those I know well, because there are also stories that there are so far out that are difficult to believe even when they reach the High Court.

It is extremely difficult to really say when they started but important to say that there are cases that have been the foundations of what is happening now because there are important facts and processes that increase the greed and arrogance of lawyers, advocates, legal consultants and ‘people in the middle’. There were stories of the operations of lawyers outside the big public and private hospitals and their ‘representatives’ and this everyone knows happened in all provinces and attorneys and their collaborators have set operations and taken over full departments because they have inside knowledge of the structures and weaknesses. Now such situations from the time they started until now are a dwarf when you compare them with the capture of the Office of the State Attorney because when such an office that is the Spiritual and Legal Gide to honesty and transparency becomes the House of Collusion then there is a collapse of money, trust, belief and good governance.

This is an office with a major legal responsibility is to provide honest, accountable, transparent, efficient fair, well-researched and informed, legal advice to all departments of the national and provincial governments. This means, alone, that the collusion irregularities with private lawyers for years led to the looting of the health coffers and budgets

A lot of senior and middle managers knew well that state attorneys did not file court papers in time, did not help the department in its litigation cases. not attend seriously to litigation matters against the Department, colluded with private lawyers and mediators, and were instrumental in settling out of court. In the corridors of the Department there were people who talked about these things because day in and day out the exorbitant financial legal demands led the Department to bankruptcy. This does not mean that the state lawyers did not appear in court, but they lost the cases. The Office and its staff were integral part of syndicates, their collaborators. The culprits thought or knew they were untouchable because the national and provincial offices of the Office were mismanaged and badly administered possibly on purpose. Those ready to blow the whistle never did because they knew they would be fired on the spot. This was and is the situation.

2.***Did the legal services passed through tenders? Were the tenders following the appropriate measures, laws rules and regulations?***

Everyone knows that all services that need to do serious work with the Department need to be selected very carefully and according to the rule of law and order and this needs to be done with one thing in mind or two things; firstly, honesty and secondly the best of the best and the cheapest.

When we acquire services or electricity with basically look for two key things in supply chain, good quality and the best value for money. In the case of our Department we come to a situation where we appoint the worst legal aids at the highest prices, and that is why it is bankrupt.

It is bankrupt because those who run it, politicians and administrative staff do not know or are not interested in the economic side of the Department that needs to be according to the rules and legislation of the country and the Treasury. This means that when we need lawyers to do a job they need to be according to the most competitive prices and be reputable and solid. They need to be in time when they are needed and to be efficient in order to win cases, be on time, able to produce quality outcomes in the cases.

What counts in every job is the success and a quality-based outcome of a given situation? We need a maximisation of the output for a given input while at the same time we keep the same quality as far as possible.

For all the years I work here the systemic gaps in the supply and procurement system have been manipulated by the corrupt group in such a way as to defeat all signs and evidence of effective planning and efforts to advance the suitability of those who win the tender. Whoever wins are pre-determined. It is a major problem.

**Interviewee 7, Senior Public Servant, KwaZulu-Natal Health Department 12 September 2018.**

1. ***How the situation in the Department started with the legal firms that created all these problems in the process?***

It started like everything else. The systems in the Department are determined and operate according to the directives of the MEC and his/her Director General. This is not because of the rules and regulations but because this is what politics are.

With the lawyers there is no difference, the big problems are related to their prices and the control they have because they are supported throughout by the Department leadership, and all these happen when the Department has staff with law degrees, there are anti-corruption sections, the structures are well organised.

The staff have their hands tied because if they talk or blow the whistles against what is happening they have no future and everyone knows and agrees that if the lawyers that are employed were better the situation would be very different, because it is not really important that a lawyer is well educated, it is important of course but the most important thing is to care about the people and the country and the department and its mission. You can find very few lawyers like this.

***2.Did the legal services passed through tenders? Were the tenders following the appropriate measures, laws rules and regulations?***

The supply chain systems exist and the evaluate tenders every day for everything under the sun including the services of lawyers and there is a Panel of Attorneys that deals directly with the legal requirements that become bigger every month but the tenders there have no quotations and it all depends on the ‘’decision makers’’.

The rules and regulations and the laws are there and need to be followed, then the Treasure inspectors come and the Auditor General’s people come to interrogate us when they are writing their report, but they themselves know what is happening. This is the reason the Auditor General’s Report are only based of figures, this happened because in the previous long, detailed reports the corrupt practices and how they took place were exposed.

Now we have lawyers on their own and also big companies who arrive here to do things that are not in Year Plans and thy are paid immediately while the problems still exist and they are not solved because the lawyers are incompetent or they do not finish the work. The Department is in serious problems because of the leadership who are responsible for leading the department forward, but they lead it to the first page of the newspapers and the social media because it is corrupt and it is involved in scandals.

They use the people in the supply chain and procurement system because they need to secure that tenders are determined by their own needs and those of the lawyers they use. There have been cases that have been exposed by the newspapers and the social media, and the lawyers’ associations have made statements and commissions of inquiry but the situation has not changed at all at least as we talk.
